# A Voxel-Map Quantitative Analysis Approach for Atherosclerotic Noncalcified Plaques of the Coronary Artery Tree

**DOI:** 10.1155/2013/957195

**Published:** 2013-11-18

**Authors:** Ying Li, Wei Chen, Kaijun Liu, Yi Wu, Yonglin Chen, Chun Chu, Bingji Fang, Liwen Tan, Shaoxiang Zhang

**Affiliations:** ^1^Institute of Computing Medicine, Third Military Medical University, Chongqing 400038, China; ^2^Department of Radiology, Southwest Hospital, Third Military Medical University, Chongqing 400038, China

## Abstract

Noncalcified plaques (NCPs) are associated with the presence of lipid-core plaques that are prone to rupture. Thus, it is important to detect and monitor the development of NCPs. Contrast-enhanced coronary Computed Tomography Angiography (CTA) is a potential imaging technique to identify atherosclerotic plaques in the whole coronary tree, but it fails to provide information about vessel walls. In order to overcome the limitations of coronary CTA and provide more meaningful quantitative information for percutaneous coronary intervention (PCI), we proposed a Voxel-Map based on mathematical morphology to quantitatively analyze the noncalcified plaques on a three-dimensional coronary artery wall model (3D-CAWM). This approach is a combination of Voxel-Map analysis techniques, plaque locating, and anatomical location related labeling, which show more detailed and comprehensive coronary tree wall visualization.

## 1. Introduction

Noncalcified plaque (NCP, referred to as “soft plaque”) [1] usually shows lower attenuation values than calcified plaque in a CT image, which has been associated with the presence of lipid-core plaques [2]. Retrospective studies have shown an association between plaques containing non-calcified components and acute coronary syndrome (ACS) [3, 4]. Therefore, it is important to detect and monitor the progress of NCPs.

According to whether or not the body has to be injured during detection of a lesion, the imaging techniques for detection and quantitative analysis of NCPs are classified into two categories: invasive methods and noninvasive methods [5]. Imaging techniques, such as intravascular ultrasound (IVUS) and optical coherence tomography (OCT), provide detailed visualization of luminal and plaque morphology and reliable quantification of the atheroma burden and its composition [5]. Although intravascular techniques have good discriminability for NCPs, they are invasive and expensive and can only be performed in proximal vessel segments [6]. Therefore, they are not appropriate to monitor the progress of NCPs of the whole coronary tree over a short time interval. Compared with intravascular ultrasound (IVUS), contrast-enhanced coronary Computed Tomography Angiography (CTA) has the advantages of being noninvasive, convenient, and economical and offers excellent diagnostic accuracy for coronary plaques [6–10]. The potential of these imaging techniques to identify atherosclerotic plaques in the whole coronary tree has raised the interest of radiologists [11]. The range of attenuation relevance to different types of plaque in CTA has been a concern over recent years. For example, there are three typical plaques that include non-calcified plaque (NCP, referred to as “soft plaque”), partially calcified plaque (PCP, also called “mixed plaque”), and calcified plaque (CP). Further details about CT attenuation value can be found in review [1].

The main limitation of traditional methods in a CTA image for the visualization of coronary artery disease is the inability to provide information about vessel walls [1]. In order to recognize NCPs, a radiologist needs to detect stenosis through various reconstruction methods and then quantitatively analyze plaques by manually drawing the boundaries of the wall and plaques [8, 10]. The current standard for coronary CT angiography plaque quantification is automatic but requires manual tracing of contours, separating epicardial fat from the vessel wall and enclosing non-calcified and calcified plaque components; this contribution promotes the plaque quantitative accuracy by accurately describing the wall border but is time consuming and may be prone to intraobserver variability [12–14]. In order to automatically trace the wall border, a previous study proposed an interactive approach that radiologists have to mark the initial and end points of the plaque in a curved multiplanar reformatted (CMPR). Further, an automated algorithm for unsupervised computer detection of coronary artery lesions has been proposed [15], but plaques are prone to be missed if they do not belong to “stenosis” by their definition. Therefore, the identification of the wall and plaques is still a challenging area.

In addition, with the development of PCI, more meaningful quantitative information is necessary to plan the path of the percutaneous coronary intervention and to assess the outcome. It is important to predict the potential location related danger in the process of PCI and also whether the catheter is able to pass through the vessel where plaque is located in the coronary tree. Higher requirements are put forward on further information of quantitative plaques such as size, type, and location quantification in a 3D space.

Above all, in order to overcome the limitations of coronary CTA and provide more meaningful quantitative information for PCI, we propose a quantitative analysis approach based on a mathematical morphology named Voxel-Map for non-calcified plaque based on a three-dimensional coronary-tree model. This method is a combination of Voxel-Map analysis techniques, plaque locating, and anatomical location related labeling that show more detailed and comprehensive coronary tree wall visualization.

## 2. Materials and Methods

### 2.1. Imaging Acquisition

All patients were scanned with a DSCT scanner (Somatom Definition, Siemens Medical Solutions, Germany). No beta-blockers were administered for the scan irrespective of the individual heart rate. The ECG was continuously recorded and stored throughout the scan. A nonenhanced DSCT was carried out from 1 to 1.5 cm below the level of the tracheal bifurcation to the diaphragm in the craniocaudal direction. For contrast-enhanced scans, intravenous bolus (60–80 mL) of a contrast agent with 370 mg of iodine per milliliter (iopromide, 370 mg of iodine/mL; Ultravist 370, Bayer-Schering, Berlin, Germany) was injected at a flow rate of 6 mL/sec, and a 50 mL chaser saline bolus was achieved with an automated injection through a power injector (Ulrich, USA). Estimation of individual circulation times was based on the test bolus technique with 20 mL bolus tracking. Data acquisition parameters for CT angiography were 0.6 mm collimation, 330 ms rotation time, 120 kV tube voltage, and 400 m as tube current. A contrast-enhanced volume data set was acquired with retrospective electrocardiogram (ECG) gating to allow reconstructions during all phases of the cardiac cycle. Transaxial images were reconstructed with 0.75 mm section thickness, 0.4 mm increment, and a medium-soft convolution kernel (B26f). The position of the reconstruction window in the cardiac cycle was individually selected to minimize artifacts.

Through Voxel-Map analysis and quantification algorithms implemented by Matlab software version 8.0 (R2012b), we treated each image as a vector. All images of one patient are treated as a set of vectors. The processing is in parallel in Matlab without complex conditions and loop operations. Segmentation, 3D reconstruction, and centerline were extracted and labeled by Amira software (V. 5.4).

### 2.2. Segmentation and 3D Coronary Tree Model

**Figure 1:**
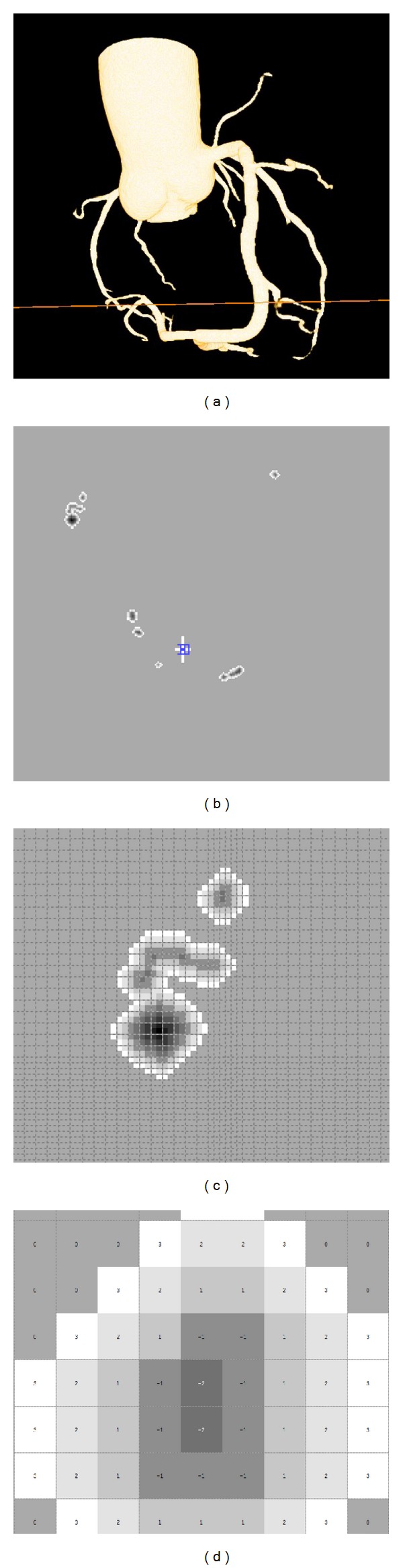
3D-CTM reconstruction and creating Voxel-Map. The reconstruction of 3D-CTM (a); the horizontal plane in the position of 3D-CTM where lined in panel a (b) and a local region of the horizontal plane (c); more detailed values of pixels are shown in Figure 1(d). Positive and negative signs represent dilation and erosion, respectively, and the values represent the distance off lumen.

According to recent pieces of literature on the attenuation cutoffs between the arterial wall and lumen [6, 10, 15–17], a voxel with an attenuation value greater than 160 Hounsfield units (HU) was defined as being the first voxel within the lumen. Based on this assumption and according to our experiment results (see Figure 1), which showed that the CT attenuation value gradient decreased from the inside to the outside the arterial wall, we can conclude that most of the voxel of the inner lumen will be greater than 160 HU. On the other hand, the attenuation values outside of lumen will be less than 160 HU. As such, we set 160 HU values as the threshold to segment highlight voxels in lumen. Then we refined the coronary tree by a region growing method to fill small holes and obtained lumen boundaries, which are well satisfied to connective relationships. After that, we obtained the segmentation of the three-dimensional coronary tree, which set 1 as foreground and 0 as background. We then used this data for centerline extraction. After an array multiply was performed on segmentation images and original CTA images, a three-dimensional coronary tree model (3D-CTM) was generated, which maintained the original attenuation values and excluded approximate attenuation values belonging to other regions. We used this model for Voxel-Map analysis.

### 2.3. Centerline Extraction

We imported the segmentation of the 3D coronary tree data into Amira software and selected “skeleton” and “centerline,” and then the 3D centerline of the coronary tree was generated automatically. After setting the segment with a minimum value of z-coordinate as root, we identified the tree as hierarchical relations and manually labeled all segments by anatomical names referring to the 17-segment model defined by the American Heart Association (AHA) [18]. We then exported the information for the centerline as a data structure which includes the start point, length, label, and the connect relationship of each segment, along with the *x*-, *y*-, and *z*-coordinates of each point and its radius.

### 2.4. Morphological Voxel-Map

We developed a Voxel-Map based on mathematical morphology [19], which is a broad set of image processing operations that process objects based on shape. The Voxel-Map includes two parts: one is dilation that reflected the voxel changes from lumen to wall, and the other is erosion that reflects the voxel changes inside the lumen.

The dilated vessel lumen by original pixel values at the morphological edge by formula is as follows:

(1) $$\begin{array}{c} A⨁B=\left\{z\;|\;{\left(\hat{B}\right)}_{Z}\cap A\neq \phi \right\},\;B=\left\{B_{1},B_{2},B_{3}\right\}, \end{array}$$

where $\hat{B}$ is the reflection of the pixel locations *B*. In other words, it is the set of pixel locations *z*, where the reflected pixel locations of *B* overlap with foreground pixels in *A* when translated to *z*. *A* and *B*, respectively, represent original lumen pixel locations, and its surrounding pixels. The boundaries forming lumen to outside are represented as *B*_1_, *B*_2_, and *B*_3_, with each point in boundary only having one pixel.

The morphological erosion of the edge returned nearest pixels of inside boundary. The operations are defined as follows with the formula

(2) $$\begin{array}{c} A\Theta B=\left\{z\;|\;{\left(B\right)}_{\text{Z}}\subseteq A\right\},\;B=\left\{B_{-1},B_{-2},\ldots ,B_{-n}\right\}. \end{array}$$

*B*_−1_, *B*_−2_, …, *B*_−*n*_ represent the 1,2,…, *n* boundaries from the border of lumen to the center, and *n* is the thickness of the lumen. The voxel with an orientation to the outer lumen has an inner wall that is defined as positive and on the contrary wall, is defined negative. After performing morphological erosion on the current lumen region each time, the region is smaller by one pixel in every direction.

### 2.5. Quantification

#### 2.5.1. Classified Attenuation Values

We divided attenuation values on the wall into six levels from one to six to describe the various severities of plaques: 0–49, 50–99, 100–199, 200–299, 300–399, and ≥400, which was extracted from the 3D-CTM and assigned a different color for each range (illustrated in Figure 6). The first three and the last three ranges describe the severity of NCP and CP, respectively. As the contrast-enhanced CTA does not offer the best images for detection of CP, which can easily be identified and quantified, we do not consider discussing CP in this paper.

#### 2.5.2. Plaques Location and Anatomical Location Related Labeling

Traditional quantification parameters can refer to pieces of literature [6, 10, 14, 15, 17, 20]. We want to emphasize on a new type of quantitative information: the plaques location and anatomical location related label, which is useful in planning pathway, guiding procession, and assessing results for PCI. The steps are as follows.

**Step 1**

A 3D surface reconstruction method was used to reconstruct non-calcified plaques (see Figure 7). Then plaque model was generated, which not only can be used in visualization in 3D space but also can be saved as “obj” format, which is a standard 3D object file format consisting of vertex's geometric position in space by *x*-, *y*-, and *z*-coordinates.

**Step 2**

Anatomical labeling: the segmentation of CTM was used as a bridge associating the 3D location of plaque with their anatomical labels, by finding the intersection set of 3D coordinates of plaque, in the segmentation of CTM, and the intersection set of 3D coordinates of the segmentation of CTM and labeled centerline tree, respectively, and intersecting the two sets.

## 3. Results

### 3.1. Voxel-Map Approach

After processing by Voxel-Map on 3D-CTM, from the outer border of lumen to the outer border of wall, four layers are labeled as −1, 1, 2, and 3, respectively, as shown in Figure 1. Any voxels whose attenuation values are less than 0 HU are considered as epicardial fat [6] and will be set as 0 Hu. If a voxel's position not in the Voxel-Map was excluded from the 3D-CTM, a 3D-CAWM was generated.

### 3.2. CT Attenuation Values

#### 3.2.1. The Difference of Attenuation Values between Vessel Lumen and the Boundary Layer of Vessel Wall

**Figure 2:**
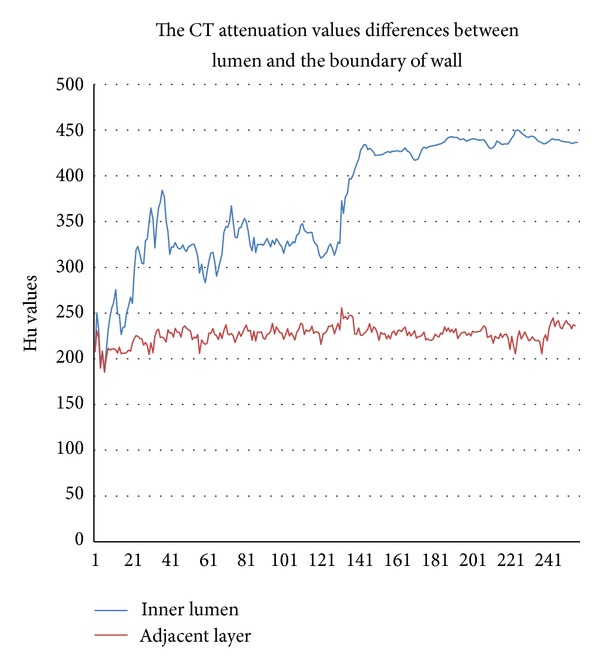
The blue and red lines represent the mean CT attenuation values of inner lumen and the border of lumen nearby wall, respectively. The *y*-axis is the range of CT attenuation values; the *x*-axis is slice number of CTA images.

The attenuation values between vessel lumen and the boundary layer of vessel wall have different characteristics, as shown in Figure 2; in a whole coronary tree, the mean attenuation values for inner lumen are sharply increasing while closing to the aorta, but for the boundary layer adjacent lumens are relatively stable. This means that different individuals might have different CT attenuation values with different doses of contrast media for the inner lumen in various positions of the coronary tree but hold relatively stable values with nearby artery walls.

#### 3.2.2. The Gradient Changes of Attenuation Values on Various Layers of Vessel Wall

**Figure 3:**
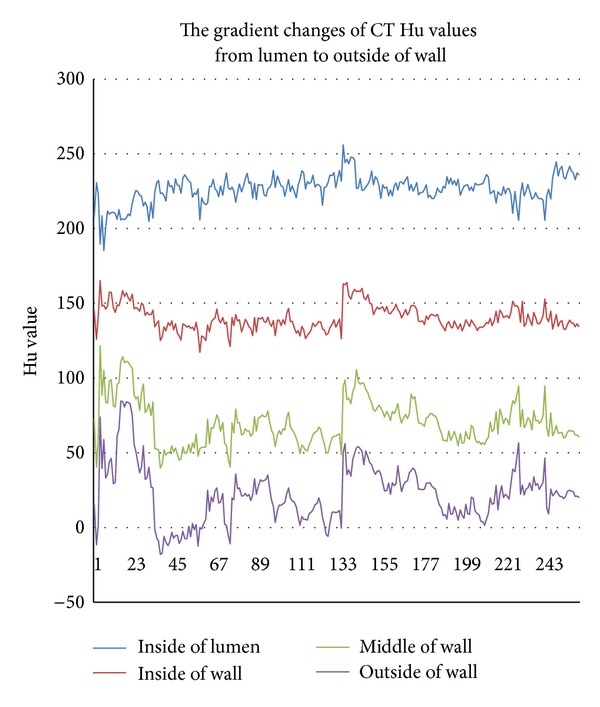
The mean CT attenuation values of various layer on wall are gradient decreased from inside to outside. The *y*-axis is the range of CT attenuation values; the *x*-axis is slice number of CTA images.

As shown in Figure 3, the mean CT attenuation values of various layers on a wall are at a gradient decreasing from inside to outside. Our experimental results show that after being dilated three times, most voxel values are equal or less than 0, which means that those voxels cross the outer borders of the wall [6], so we set the maximum as B3 and set the negative values included in the wall as 0. Artery walls were divided into three layers: inside, middle, and outside. The reason we divided attenuation values on the wall into six levels to describe the various severities of plaques is also based on the gradient distribution of attenuation values. As shown in Figure 3, the first three levels, 0–49, 50–99, and 100–199, included most of voxels in each layer, respectively. The attenuation values of vessel walls on the range of the last three levels, 200–299, 300–399, and ≥400, should be considered as calcified plaques with the various severities. The results of attenuation values belonging to various levels are shown in the right column of Figure 6.

### 3.3. Quantitative Analysis

#### 3.3.1. Anatomical Labeling

**Figure 4:**
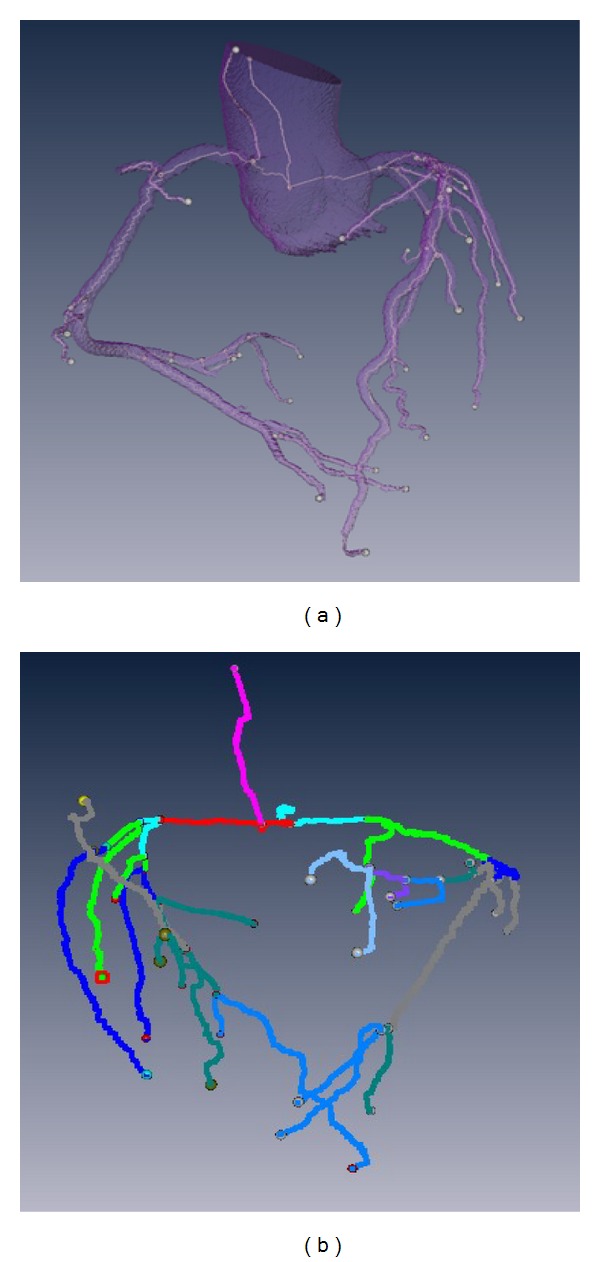
Centerline extraction (a) and anatomical labeling (b).

The result is that centerline extraction of 3D-CTM, as shown in Figure 4(a), was organized by tree graph, and Figure 4(b) shows the resulting anatomical labeling based on the tree graph. The different colors represent different segments. Once we obtain the *x*-, *y*-, and *z*-coordinates of plaque, the computer will obtain which label the segment belongs to.

#### 3.3.2. The 3D-CAWM Analysis

**Figure 5:**
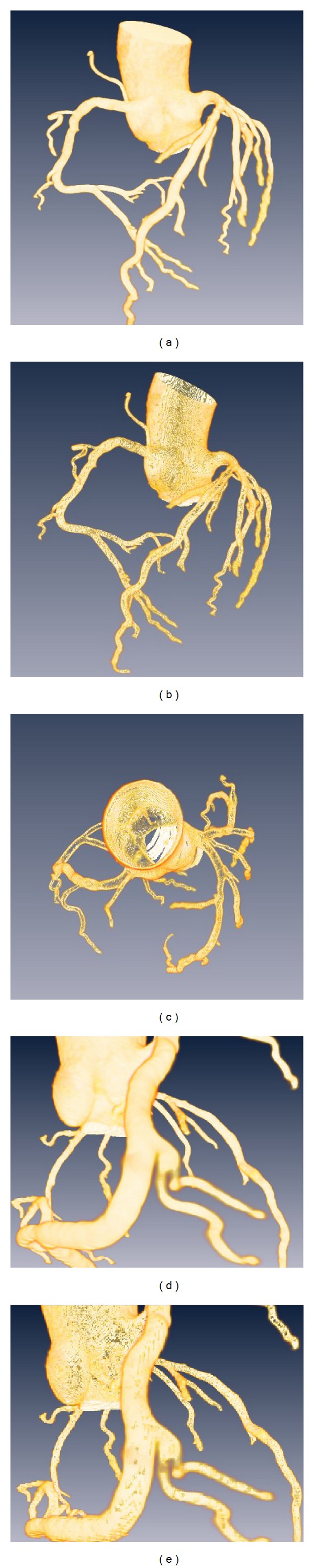
The three-dimensional coronary tree model (3D-CTM) and coronary artery wall model (3D-CAWM). The original 3D-CTM (a); the 3D-CAWM (b); the top view of 3D-CAWM (c); the stenosis of coronary tree in 3D-CTM (d) and 3D-CAWM (e).

**Figure 6:**
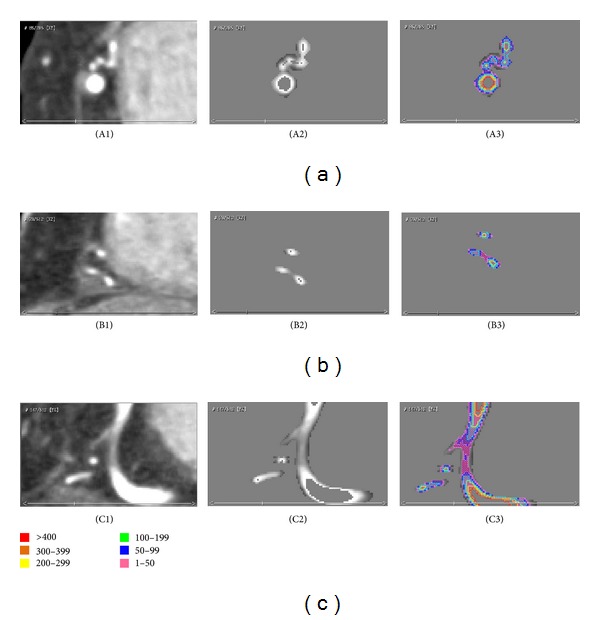
Quantitative analysis of 3D-CAWM. The left column shows that the original CTA images, A1, B1, C1, are horizontal plane, sagittal plane, and coronal plane, respectively. The middle column figures of A2, B2, and C2 show the corresponding planes in 3D-CAWM, and A3, B3, and C3 show the corresponding quantitative results based on Voxel-Map.

The result of 3D-CAWM, as shown in Figure 5, provided the shape and details of the whole coronary artery wall including the proximal, middle, and distal segments. Compared with the original 3D-CTM, the output of the 3D-CAWM processed by a Voxel-Map focusing on the morphology and details of the wall and the stenosis is more remarkable. On contrast-enhanced CTA images (the left column in Figure 6), the lumen can be easily identified as areas of high attenuation, while it is difficult to identify the wall and plaque. Compared with original images, the 3D-CAWM (the right column in Figure 6), after having the Voxel-Map applied, is able to provide details of the nearby wall, and the different colors represent different severities.

### 3.4. Plaques Visualization and Location

**Figure 7:**
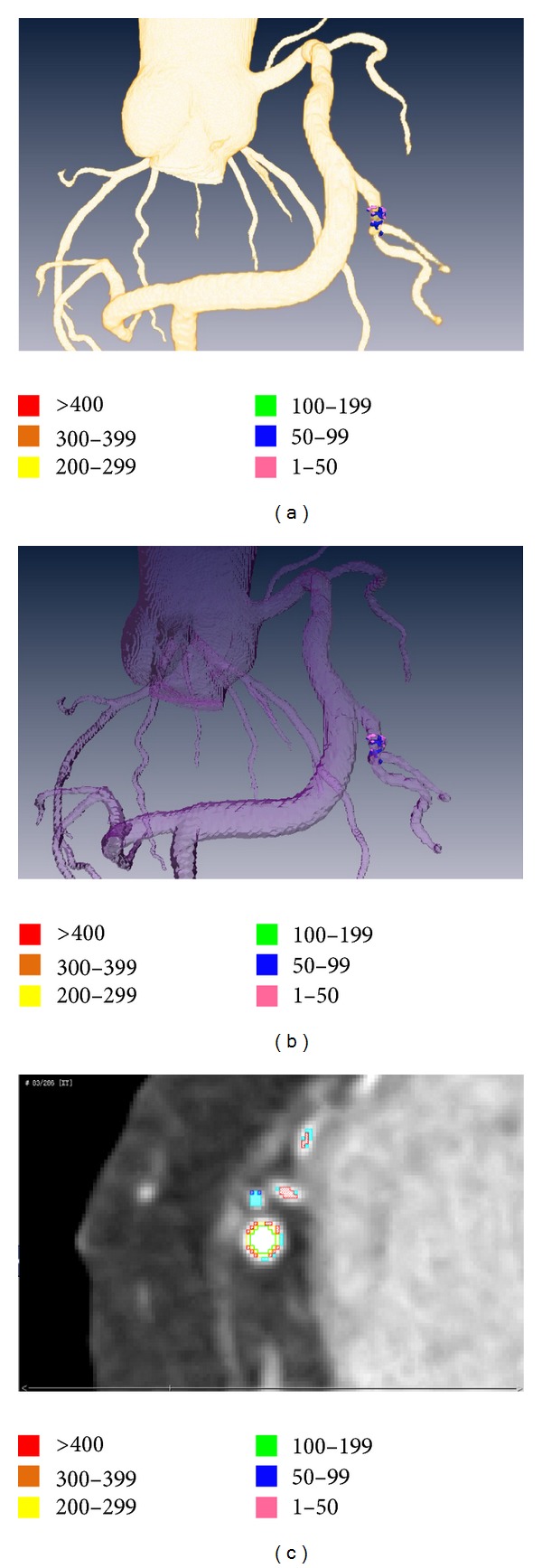
Plaques visualization. The reconstruction of coronary tree and NCPs (a); the transparent coronary tree to observe plaques (b); the analysis results in horizontal plane (c). The different colors represent different severities.

The visualization of plaques in 3D-CAWM is shown in Figure 7. The locations of various NCPs on the coronary tree are recorded by computer automatically in the form of *x*-, *y*-, and *z*-coordinates. At the same time, anatomical labeling of plaque is generated by comparing these coordinates to the coordinates of the centerline in the 3D-CTM that were assigned an anatomical label.

**Figure 8:**
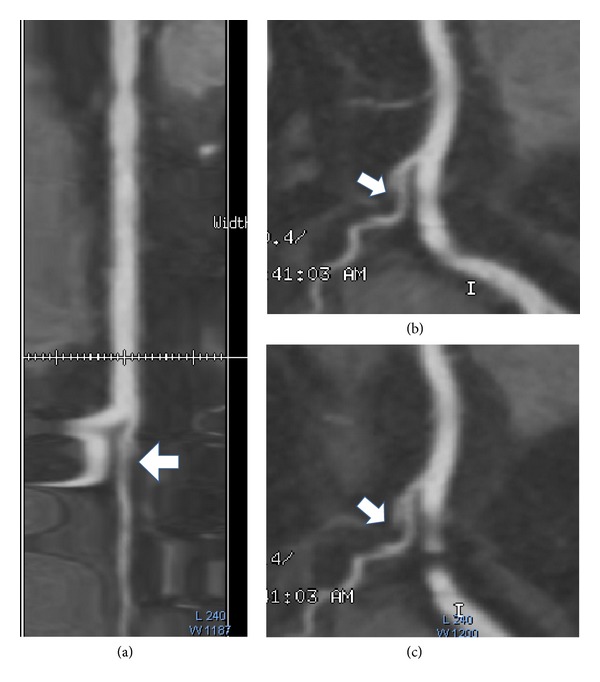
CMPR vessel representation (by AW Volume Share 4 software, GE, America). Longitudinal straightened views (a); the left rotated 284 degree views (b) and the left rotated 295 degree views (c).

The computer analysis results showed that the plaque consisted of level 1 and level 2, and the volume and percent of each level of plaque were 0.9834 mm^3^ and 33.29%, 1.9703 mm^3^ and 66.71%, respectively, and the location label belongs to RMA (right marginal artery). The location results of stenosis are consistent with the CMPR review results by an experimental radiologist. Compared with the stenosis views in CMPR (shown in Figure 8), the characteristic of NCPs is more directly related to the results of quantitative analysis.

## 4. Discussion

### 4.1. Voxel-Map's Function

The key feature in quantitative analysis of NCP is recognition of artery walls. Manually describing the wall and plaque by a radiologist is timeconsuming and may be prone to intraobserver variability [12–14] that influences the accuracy of quantitative analysis. In traditional automatic quantitative methods, the reader marks starting and ending positions of the plaque in the CMPR and then displays and adjusts attenuation thresholds for NCP and CP [13, 21], which is based on the hypothesis that before you quantitatively analyze plaques, you must first find their wall border. The positions of the start and end marked by reader also influence the computation of the mean attenuation values in various types. In order to solve this problem, we developed a Voxel-Map based on mathematical morphology [19] to structurally and quantitatively analyze coronary artery walls on a 3D-CTM, which detects walls in a similar approach as IVUS but overcomes its limitation of only being able to be performed in proximal segments [6].

### 4.2. The 3D-CAWM Analysis

A 3D-CTM only includes the HU values of lumen and thus does not satisfy the need to analyze NCPs, as the attenuation values of NCPs are lower than the lumen,which were often excluded in the process of segmenting for 3D-CTM. Compared with original 3D-CTM, the stenosis is more remarkable on the 3D-CAWM that was processed by the Voxel-Map. The analysis method focuses on the morphology and details of wall, which can directly show the composition of plaque attenuations that are associated with its severity. The advantage of 3D-CAWM is that it can analyze the details of distal vessels and provide meaningful information for a radiologist's diagnostic decision, which is impossible in previous methods. The obtained 3D-CAWM allows comprehensive visualization of vessel geometry and plaque distribution and can be further used in research to study the association between local plaque types and the progression of atherosclerosis.

### 4.3. Attenuation Values of Plaque

Recently, many studies had shown different ranges of the attenuation values in various compositions of plaque [6, 10, 14, 15, 17, 20]. However, according to previous studies, even IVUS has some limitations in the assessment of the true composition and vulnerability of plaque, due to substantial overlap of the corresponding attenuation values [17, 21]. As such, we further subdivided attenuation values on the wall into six levels to describe the various severities of the plaques. The outliers that located within one layer but not belonging to its level might be considered as various severities of NCPs, which should be paid more attention by radiologist. Our group proposed the Voxel-Map as a new approach for analyzing and deep understanding attenuation changes in different degrees on the vessel wall in 3D space. Using our approach, early diagnosis and process monitor of the slight NCPs in vessel walls for patients become possible.

### 4.4. Plaques Location and Anatomical Location Related Label

Through the 3D surface reconstruction of non-calcified plaques, we can directly observe the location of plaque in the coronary tree and also can recognize its composition (see Figure 7). Information about the locations that have plaques and to which branch they belong benefits doctors and will improve the success rate of percutaneous coronary intervention by evaluating the degree of danger and allowing for planning the pathway before operation and for assessing the outcome after operation. An anatomical location-related label is linked to plaque location, and its properties, such as per artery or per segment, can be used for reporting pathological findings according to CCTA image guidelines by radiologists and cardiologists [22, 23].

## 5. Conclusions

We proposed a Voxel-Map quantitative analysis approach, overcame the drawbacks of a CTA image regarding analysis of non-calcified plaque, and provided information regarding vessel walls based on a 3D coronary tree model. In this paper, we presented the Voxel-Map design and related quantitative analysis. The approach we proposed can provide details about the morphology from lumen to outer wall border, the types of plaques, and its location. This noninvasive, convenient, and economical approach can also be used for advantage of asymptomatic patients and to identify predictors of future cardiovascular events. Furthermore, it can be used in planning and assessing the outcome of percutaneous coronary intervention.
